# Efficacy comparison of the anterior low small incision and the traditional incision for treatment of thyroid adenoma

**DOI:** 10.12669/pjms.305.4767

**Published:** 2014

**Authors:** Zhen Zhang

**Affiliations:** Zhen Zhang, Department of Surgery, Mengyin People’s Hospital, Shandong Province, 276200, P. R. China.

**Keywords:** Efficacy, Small incision, Thyroid adenoma

## Abstract

***Objective:*** To compare the clinical efficacy and surgical effects of the anterior low small incision and the traditional incision in the treatments of thyroid adenomas.

***Methods:*** Eighty cases of thyroid adenoma patients were randomly divided into a control group and an experimental group. Forty patients in the control group received the traditional incision, whereas the experimental group received the anterior low small incision treatment. The clinical efficacy and complications were compared between these two groups.

***Results:*** The surgeries were all successfully completed. The surgical blood loss, incision length, length of stay, patient satisfaction and health care costs in the experimental group were significantly better than those in the control group, with differences statistically significant (P < 0.05).

***Conclusions:*** The advantages of the anterior low small incision in the treatment of thyroid adenoma patients include smaller incision, lower costs, and fewer complications, when compared with the traditional incision.

## INTRODUCTION

Thyroid adenoma is one of the most common benign thyroid tumors,^1^ accounting for about 70%-80% of the total number of thyroid tumors. Thyroid adenoma is characterized by a follicular differentiation and complete encapsulation. The prevalence of thyroid adenoma varies greatly between males and females with an approximate ratio of 1 to 6.^2^^-^^4^ Currently, surgical excision is the major method in the clinical treatment of thyroid adenoma.^5^^-^^8^ However, such traditional surgical incision usually resulted in relative long length of incisions, large amount of bleeding and many other complications. Furthermore, the traditional surgical incision easily generates visible scars that are permanently left on the patient’s neck and seriously affects the life quality of patients.^9^ Therefore, many patients, especially the young female patients, often disagree to have the traditional surgery for the treatment of thyroid adenoma.

In this study, 80 cases of patients with thyroid adenoma receiving surgeries of removal of thyroid adenoma were analyzed. Among them, 40 patients were treated with the traditional surgery for the removal of thyroid adenoma, and the other 40 patients were treated by the anterior low small incision for the removal of thyroid adenoma. The clinical efficacy was compared in these two groups and the results are reported as below.

## METHODS

***Patient data: ***Eighty patients with benign thyroid tumors were enrolled in this study in our hospital between January 2010 and January 2013. The anterior neck tumors were diagnosed, with diameters of 1.4 cm - 4.1 cm and the average diameter of 2.3 ± 1.5 cm. All patients got thyroid ultrasound done and thyroid function and CT examination. The cytology examination results suggested that all were benign lesions. No absolute contraindication for surgery existed related to the vital organs such as heart, lung and kidney. In this study, 80 patients were randomly grouped into the control group and the experimental group, with 40 cases in each group. General information between these two groups such as ages, gender, conditions of the disease and tumor diameters all showed no significant statistical difference (P > 0.05).

Prior written and informed consent was obtained from every patient and the study was approved by the ethics review board of Mengyin People’s Hospital.

***Surgical treatments: ***The patients in the control group received traditional incision for the removal of thyroid adenoma. The incision site was chosen about 2cm above the acromioclavicular joint. High-frequency electric knife was used to separate the loose connective tissues between platysma and cervical fascial space. Neck white line was then longitudinally incised. Anterior muscle were appropriately retracted by external force to expose thyroid adenoma. Thyroid lobe was finally drifted away carefully. The recurrent laryngeal nerve was carefully protected during the surgery. Intra operative frozen examination was performed to confirm the adenoma. Complete hemostasis and suture of the incision were done after the surgery. Silicone drainage tube was placed under the incision with slight pressure and was then carefully bandaged.

In the experimental group, the anterior low small incision was applied for the removal of thyroid adenoma. The location of incision was then chosen 2 cm above the sternum and 3 - 4 cm small incision was given along the skin crease. The high-frequency electric knife was used to open the subcutaneous tissue and platysma, and the neck white line was then longitudinally incised along the neck midline. The thyroid envelope was drifted to the top of the incision to fully expose the thyroid. The high frequency electric knife was used to cut the thyroid tumors directly, at 0.2 to 0.5 cm edge of the thyroid tumors. Intraoperative frozen examination was performed to confirm the adenoma. Complete hemostasis and suture of incision were done after the surgery. Silicone drainage tube was placed under the incision. The non-absorbable intradermal suture line was used to suture skin incision with slight pressure and was then carefully bandaged.

***Experimental indicators: ***In this study, indicators were compared in the two groups, including the patient's operative time (time from skin incision to complete wound suture), blood loss (by the addition of the amount of suction and swab of gauze), drainage (total amount of liquid in drainage bag), length of hospital stay (days of stay after surgery), beauty score of incision (self-satisfaction ratings by patients after receiving the stitches; score 1 as the worst, score 10 as the most satisfactory) and the degree of satisfaction, etc. The rate of complications in patients was examined such as incision adhesions, wound infection, cervical pain, neck tightening and difficulty in swallowing. The standards to evaluate clinical efficacy were: (i) healed: clinical symptoms and signs disappeared completely after the surgery without any recurrence after one year follow up; (ii) improved: the clinical symptoms and signs basically disappeared after the surgery with no recurrence within 3 months after surgery; and (iii) ineffective: breathing difficulties were relieved after tracheotomy, but it was difficult to do surgical resection.

***Statistical analysis: ***SPSS 13.0 software was used for the statistical analysis. Frequency (n) was used to count data and chi square was used for the test. The mean ± standard deviation ($\bar{X}$ ± S) was used to measure data. Set data and paired data were applied in the study for the test.

## RESULTS

***Anterior low small incision results have much better effects than the traditional incision surgery:*** The patients in the control group and the experimental group received the traditional incision surgeries and the anterior low small incision to remove thyroid adenomas respectively. The indicators in the two groups were compared, including the time taken for surgery, amount of bleeding, drainage, length of hospital stay and incision beauty score. The indicators in the two groups were statistically analyzed and the results showed statistical difference (P < 0.01), which is shown in Table-I. As shown in Table-I, the amount of bleeding in the experimental group was 8.8 ± 1.7 ml, whereas the amount of bleeding in the control group was 20.6 ± 5.5 ml. Incision beauty score in the experimental group was 8.8 ± 1.2, but the incision beauty score in the control group was 2.1 ± 1.4.

**Table-I:** Comparison of surgical conditions in the experimental group and the control group ($\bar{X}$±S).

***Groups***	***No. of patients*** ***(n)***	***Surgery time*** ***(min)***	***Amount of bleeding (ml)***	***Drainage***	***Days of hospital stay (d)***	***Incision beauty score*** ***(%)***
Experimental group	40	46.3±10.2	8.8±1.7*	25.3±8.6*	3.2±0.4*	8.8±1.2*
Control group	40	43.2±11.5	20.6±5.5	50.6±15.5	7.8±1.7	2.1±1.4

*, Note: p < 0.01, compared with the control group.

As shown in Table-II, in the 40 cases, 38 patients were healed and 2 patients were improved after the surgery in the experimental group. The total effective rates were 100% and the satisfaction rates were 100%. However, in the control group, only 31 cases of the total 40 cases were cured. The total effective rate was 95% and the satisfaction rate was 77.5%. These results in Table-I and Table-II suggest that anterior low small incision results has much better effects than the traditional incision surgery.

**Table-II:** Clinical efficacy and degree of satisfaction in the experimental group and the control group (%).

***Groups***	***No. of patients (n)***	***No. of healings (n)***	***No. of complications (n)***	***No. of patients with no effect (n)***	***Overall ratings of efficacy (%)***	***Overall ratings of satisfaction (%)***
Experimental group	40	38	2	0	100*	100*
Control group	40	31	7	2	95	77.5

* Note: , p < 0.05, compared with the control group.

***Comparison of incidence of complications in the experimental group and the control group:***

To compare the complications in the two groups, the 1-year follow up investigation was performed. Postoperative complications, including incision adhesions, wound infection, cervical pain, neck tightening, difficulty in swallowing, were investigated (Table-III). The overall incidence of surgical complications was 2.5 % (1/40) in the experimental group, since only 1 case had neck tightening diagnosed. However, in the control group, 3 cases were found to have surgical adhesions, while 2 cases had neck tightening. In addition, difficulty in swallowing (1 case), wound infection (1 case), and pain of cervical area (1 case) were found. The total incidence of surgical complications in the control group was 22.5 %. The difference in the two groups showed statistical significance (P < 0.01), suggesting that patients in the anterior low small incision group have lower incidences of complications than those in the traditional incision surgery group.

**Table-III:** Comparison of complications in the experimental group and the control group ($\bar{X}$ ± S).

***Groups***	***No. of patients*** ***(n)***	***Incision adhesion*** ***(n)***	***Wound infection (n)***	***Pain of cervical area (n)***	***Neck tightening (n)***	***Inflexible swallowing (n)***	***Overall ratings of incidence (%)***
Experimental group	40	0	0	0	1	0	2.5*
Control group	40	3	1	1	2	1	22.5

*, Note: p < 0.01, compared with the control group.

## DISCUSSION

There are several ways of surgical treatments for thyroid adenoma and such treatment has been developed rapidly.^5^^-^^8^ However, the traditional surgery is difficult to be accepted by the young women due to 6-8 cm surgical scarring and even inevitably permanent scarring on the neck after the surgical treatment of thyroid adenoma.^10^ Low anterior small incision for the treatment of thyroid adenoma has emerged as a new surgical method.^11^ The surgical incision in the low anterior small incision is designed according to the length of patients’ neck and location of the lesion, without widely separating the platysma under plane and breaking away from the strap muscles. The surgery has the effect of minimally invasive surgery based on the healing of disease. This makes the surgical incision hidden and miniaturization without leaving any visible scars and thus attaining good cosmetic results. The results in this paper indicate that the low anterior small incision for the resection of thyroid adenoma is more effective than the traditional incision out of consideration of cosmetic results.

Many methods can be applied in the treatment of benign thyroid adenomas. Recently, the minimally invasive treatment method assisted by endoscopic treatment has been developed rapidly.^12^ However, such surgery increases the incidence of long-term postoperative complications due to the application of aggressive lobe resection.^13^ The surgeons should always maintain a clear operative field and skilled endoscopic manipulation techniques. In addition, they should manipulate the endoscopy more flexibly in order to precisely and carefully distinguish anatomical structures and successfully achieve proficiency of tumor removal and hemostasis. In addition, the application of endoscopic thyroidectomy also has some complications, such as parathyroid damage, thyroid dysfunction, laryngeal nerve bleeding, recurrent laryngeal nerve injury and postoperative bleeding, etc. In comparison with traditional surgery, endoscopic treatment for the resection of thyroid adenoma has unique complications mainly including subcutaneous emphysema, chest wall skin irritation, and diffusion etc. Meanwhile, the endoscopic treatment need the high costs and leads to long operative time.^14^

In this study, the total efficacy was 100% in the experimental group and the satisfaction rate was also 100%. However, the total efficacy was 95% in the control group with a satisfaction rate of 77.5%. The clinical efficacy and satisfaction rate of the experimental group was significantly better than the control group. The overall incidence of surgical complications in experimental group was 2.5%, while surgical complications was 22.5% in the control group, indicating that the difference in the two groups was statistically significant.

The low anterior small incision has advantages of good curative effects and the high efficacy. In addition, the low anterior small incision for the treatment of benign thyroid tumor results in safe, effective, and clear operative field. Meanwhile, the ratings of risk of complications are decreased in the experimental group, such as the recurrent laryngeal nerve injury, parathyroid glands injury and the risk of damage to blood vessels and trachea.^15^ Altogether, the low anterior small incision for the treatment of benign thyroid tumors has obvious advantages, such as less invasive, quick recovery, good cosmetic results, effective clinical results and fewer complications. 
